# Do poor patients suffer from inaccurate diagnoses more than well-to-do patients? A randomized control trial

**DOI:** 10.1186/s12909-019-1805-6

**Published:** 2019-10-22

**Authors:** Ibrahim Al Alwan, Mohi Eldin Magzoub, Ali Al Haqwi, Motasin Badri, Sarah M. Al Yousif, Amir Babiker, Sílvia Mamede, Henk G. Schmidt

**Affiliations:** 1 Department of Pediatrics, College of Medicine, King Saud bin Abdulaziz University for Health Sciences, Riyadh, Saudi Arabia; 2 College of Medicine and Health Sciences, United Arab Emirates University, Al Ain, United Arab Emirates; 3 Department of Family Medicine, College of Medicine, King Saud bin Abdulaziz University for Health Sciences, Riyadh, Saudi Arabia; 4 College of Public Health and Health Informatics, King Saud bin Abdulaziz University for Health Sciences, Riyadh, Saudi Arabia; 5 Department of Medical Education, College of Medicine, King Saud bin Abdulaziz University for Health Sciences, Riyadh, Saudi Arabia; 6 Department of Pediatrics, King Abdulaziz Medical City, Ministry of the National Guard- Health Affairs, and College of Medicine, King Saud bin Abdulaziz University for Health Sciences, Riyadh, Saudi Arabia; 7 Institute of Medical Education Research Rotterdam, Erasmus Medical Centre, and Department of Psychology, Erasmus University of Rotterdam, Rotterdam, The Netherlands; 8 Department of Psychology, Erasmus University Rotterdam, Rotterdam, The Netherlands

**Keywords:** Medical errors, Clinical reasoning, Poor, Rich

## Abstract

**Background:**

Poor patients have greater morbidity and die up to 10 years earlier than patients who have higher socio-economic status. These findings are often attributed to differences in life-style between groups. The present study aimed at investigating the extent to which physicians contribute to the effect by providing relative poorer care, resulting in relative neglect in terms of time spent with a poor patient and more inaccurate diagnoses.

**Methods:**

A randomised experiment with 45 internal medicine residents. Doctors diagnosed 12 written clinical vignettes that were exactly the same except for the description of the patients’ socio-economic status. Each participant diagnosed four of the vignettes in a poor-patient version, four in a rich-patient version, and four in a version that did not contain socio-economic markers, in a balanced within-subjects incomplete block design. Main measurements were: diagnostic accuracy scores and time spent on diagnosis.

**Results:**

Mean diagnostic accuracy scores (range 0–1) did not significantly differ among the conditions of the experiment (for poor patients: 0.48; for rich patients: 0.52; for patients without socio-economic markers: 0.54; *p* > 0.05). While confronted with patients not presenting with socio-economic background information, the participants spent significantly less time-to-diagnosis ((for poor patients: 168 s; for rich patients: 176 s; for patients without socio-economic markers: 151 s; *p* < 0.01), however due to the fact that the former vignettes were shorter.

**Conclusion:**

There is no reason to believe that physicians are prejudiced against poor patients and therefore treat them differently from rich patients or patients without discernible socio-economic background.

## Background

Poorly educated people are generally in poorer health than the better educated [1–5]. They engage in unhealthy behaviors, such as smoking, alcohol consumption, and physical inactivity [3], are suffering from stress to a larger extent [6–8], and consume unhealthy food more often [9]. In addition, people in the most-disadvantaged socio-economic-status quartile have an almost three-fold increased risk of mortality relative to those in the least-disadvantaged quartile [3]. On average, poor people die up to 10 years earlier than their more well-to-do counterparts [2].

Studies in search of causal factors have concentrated on the lifestyle factors mentioned above. Less is known yet about institutional factors that contribute to poor health, such as differential access to health care facilities [10], or late-stage diagnosis of disease [11]. Even less is known about how *physicians* deal with poor, as opposed to well-to-do, patients. It is possible that part of the greater morbidity and mortality of poor people is due to relative neglect of their doctors.

There are some studies that seem to imply that this is indeed the case, for instance in referral to psychotherapy [12], or in the diagnosis of late-stage breast cancer [13]. Rathore, et al. [14] found that poor patients were treated less adequately when acute myocardial infarction was concerned. These studies were however observational in nature. We only came across two studies in which social class was experimentally controlled. In the first study [15], participating primary care doctors viewed two video-vignettes of a scripted consultation in which the patient presented with standardized symptoms of coronary heart disease. Videotapes were identical apart from varying patients’ gender, age, class and race. Gender of patient significantly influenced doctors’ diagnostic and management activities. However, there was no influence of social class, neither on the doctor’s diagnosis, nor on the management activities undertaken. It is however possible that the actors playing the various roles were not able to present sufficiently different personalities. In a second study [16], our own research group presented residents in internal medicine with identical case vignettes for diagnosis. However, half of the subjects received the vignettes accompanied by a picture of a clearly poor person—clothes looked second-hand and contained rips; faces were dirty—while the other half received a picture of the same person, but in clearly well-to-do clothing. The expectation was that poor-looking patients would receive less attention (as measured by the amount of time needed to reach a diagnosis) and would be subject to less accurate diagnosis. However, we failed to find such differences.

In hindsight, the latter study had three potential shortcomings. The first was that we only presented two patients—a man and a woman in two different guises-- rather than a more diverse group of patients. The second was that a more or less classless version of our patients was not included in the design. It is possible that social class information—whatever its nature--influences diagnostic decision making mainly by increasing the number of possible alternative diagnoses. For instance, a poor miner plagued with headaches may lead the doctor to inappropriately think of lung-related diseases, whereas the presentation of an obese businessman with headaches may lead him or her to think of cardiovascular causes. A presentation of the same patient without class-related characteristics may not be burdened by such potentially unproductive hypotheses. And the third shortcoming was similar to the Arber, et al. [15] study, in that the patients still may have looked too much alike. It is for instance possible that doctors deduce social class from characteristics other than poor clothing or dirt on faces. We therefore, decided to follow a strategy that we successfully employed in two earlier studies on the effect of difficult patients on diagnostic decision-making [17, 18]: we did not show videos or pictures of the patients, but described them.

This article reports results of an experiment in which physicians were confronted with 12 vignettes of patients of different socio-economic status but with the same symptoms and the same underlying diseases. Using a within-subjects design, each participant was confronted, in random order, with four poor, four rich, and four patients for whom no class information was available. The number of accurate diagnoses, and time needed to reach a diagnosis, were recorded.

## Method

### Participants

Participants were 45 internal medicine residents (mean age = 28.91; standard deviation = 2.45; 31 male) from the College of Medicine, King Saud bin Abdelaziz University for Health Sciences, Jeddah, Saudi Arabia. The residents had on average 3.60 years of post-medical-school experience (standard deviation = 1.90). All 60 residents of this group were invited to participate in the study between December 2012 and March 2014, and volunteers were recruited. No incentive was provided for participation. The ethics review committee from King Abdullah International Medical Research Center (KAIMRC), approved this study. As the nature of the experiment prevented disclosure of its objectives beforehand, participants were informed about their tasks and debriefed later. All participants signed consent to use their data.

### Materials

Twelve clinical cases, prepared by one of the senior researchers (M.E.M.) and based on cases used in previous studies [17–19], were employed in this experiment. All cases had a confirmed diagnosis and consisted of a brief description of a patient’s history, complaints, symptoms, and findings from physical examination and tests. Table 1 contains the 12 diagnoses.

**Table 1:** Diseases involved in the study; descriptions of rich and poor patients

Cases	Rich version	Poor version
Stomach Cancer	The first patient of the day is a 74-year-old businessman. He is married and lives in the Al Shati locality in Jeddah. You noticed him even before he entered your office because of his luxurious car that you have seen through the window of the clinic.	The first patient of the day is a 74-year-old unemployed man. He is married and lives in Al Kirinteana Locality in Jeddah. You notice the bad smell that comes from his dirty clothes when he enters your office.
Vitamin B12 deficiency	A 62-year-old man who is a Minister and an owner of a big mall accompanied by two security guards comes to your clinic	A 62-year-old man who lives in the Gholail locality in Jeddah, working as a school guard, living in a two-bedroom house with his wife and 10 children between ages 5 and 21
Pulmonary thromboembolism	A 31-year-old lady, the second wife of the owner of the largest company in Jeddah. She is well-dressed, wearing a pure golden watch	A 31-year-old divorced lady living with her large family in Inaikish locality. The family includes six single sisters, two unemployed brothers, her retired father and blind mother.
Celiac disease	A 29-year-old woman, the chair of the association of women in business	A 29-year-old lady lives in the Lilosix area in Jeddah, works as a housemaid, and is poorly dressed
Acute viral pericarditis	A 78-year-old man who owns a five-star hotel in Mecca, lives in a palace located in Al Basatean locality in Jeddah with his four wives	A 78-year-old homeless man, whom you observed several times begging at the busiest traffic light on the way to your hospital,
Acute myeloid leukemia	A 29-year-old engineer who owned the largest construction company in the city, wearing a bisht	A 29-year-old driver, a father of six children, living in the Al Sabeel area in Jeddah.
Acute bacterial endocarditis	You have been called by the director of the hospital to take extra care of a 27-year-old businessman, well-known in town	A 27-year-old man working as a cleaner and living with his large family in Inaikish, one of the poorest areas of Jeddah
Sarcoidosis	A 25-year-old man, the eldest son of the owner of a large profitable communication company,	A 25-year-old man, unemployed and homeless, originating from Al Khumra area in Jeddah
Acute appendicitis	A 23-year-old female, the daughter of the Secretary General of Jeddah municipality, accompanied by two housemaids and a driver in addition to her mother	A 23-year-old female, the daughter of a school guard, who came walking all the way to the hospital
Community-acquired pneumonia	The patient is an obese 56-year-old woman, mother of multiple children, married to a renowned businessman in Jeddah	The patient is an obese 56-year-old woman, mother of multiple children, and the wife of a teaman (Sabbab) living in Bani Malik area in Jeddah
Liver cirrhosis	A 52-year old eminent lawyer who is also the legal advisor of the largest construction company in the city	A 52-year-old retired laborer in the civil service who is a single father of six daughters and two younger sons who are still in school
Addison Disease	A 45-year-old woman, well-dressed and wiring expensive jewelry and large heavy golden bracelets	A 45-year-old woman, married to a gardener, working in one of the parks in Jeddah with large family
Inflammatory bowel disease	A 32-year-old woman living in Al Salamah Locality in Jeddah	A 32-year-old housemaid

In each case, a few sentences described aspects of the patient’s socio-economic status. These sentences portrayed either a patient of apparently high socio-economic position, a patient of apparently low socio-economic position, or a patient without any socio-economic markers (called “rich,” “poor,” and “neutral” patients from here), effectively producing three versions of the same clinical case. Two co-authors (I.A.; M.E.M.) prepared the descriptions based on the kind of patients one would see in the consulting room in Saudi Arabia. Table 1 also contains the short descriptions of rich and poor patients included. The neutral patient description only contained age information. In all other respects the different versions were identical, leading to the same diagnosis. Table 2 presents an example of three versions of the same case.

**Table 2:** Example of three versions of a clinical case

Rich version	Poor version	Neutral version
The first patient of the day is a 74-year-old businessman. He is married and lives in the Al Shati locality in Jeddah. You noticed him even before he entered your office because of his luxurious car that you have seen through the window of the clinic.	The first patient of the day is a 74-year-old unemployed man. He is married and lives in Al Kirinteana Locality in Jeddah. You notice the bad smell that comes from his dirty clothes when he enters your office.	The first patient of the day is a 74-year old man.
	He has had complaints of slight pain in the epigastric area, anorexia and progressive weight loss in the past 4 months. He started to get fatigued easily and to have dizziness when walking, over the past 2 days. He refers occasional dark stools. The patient is a smoker. He refers chronic use of NSAID for osteoarthritis of the knees. Family history: father had a gastric ulcer. Physical examination: Patient considerably emaciated. Bp 135/80 mmHg; Pulse 88/ min.; Respiration 24/ min.; Temp 37.4 degrees of C. Heart: no abnormalities. Lungs: no abnormalities. Abdomen: slight pain on palpation in the epigastric area. Lab tests: Hb: 8.4; Ht: 20.9%	

### Procedure

The study employed a within-subjects design. A full within-subjects design would imply the presentation of all three versions of *each* case to the participants. Such presentation of three versions of the same case would however likely lead to carry-over effects: when one has seen one version, diagnosing the second, or a third, may become easier. An alternative is to present to each participant *one-third* of the cases in rich, one-third in poor, and one-third of the cases in neutral format, however in different combinations. In other words: Every participant received four rich patients, four poor patients, and four neutral patients such that all 12 diseases were seen once. For instance, if A1 represents a rich-patient version of diagnosis A, A2 its poor version and A3 its neutral version, then the first participant would receive the cases A1, B2, C3, D1, E2, and F3, etc., whereas the second participant would receive the cases A2, B3, C1, D2, E3, and F1, etc. Such *balanced within-subjects incomplete block design* enabled us to compare mean diagnostic performance scores and time-to diagnosis under the three experimental conditions.

The cases were presented on a computer screen using Qualtrics software (Qualtrics XM Platform™) First, they were informed that the study aimed to better understand the nature of clinical problem-solving in Internal Medicine. Second, they were informed that their responses were anonymous since no identifying information would be collected and that their results would have no implications for their work. Their task was to diagnose the clinical cases presented shortly. All cases were based on real patients and had a confirmed diagnosis.

Further, they were asked to work as quickly as possible; suggesting that a first impression is often correct. They should, however, not compromise accuracy. They were instructed to type only one complete and precise diagnosis which they found to be the MOST accurate for the case presented. They were also informed that once they clicked to the next case, they could not go back to previous screens. After being informed, they received a practice case, unrelated to the hypotheses tested.

### Data analysis

The accuracy of participants’ diagnoses was evaluated by considering the confirmed diagnosis of each case as a standard. Two physicians (I.A.; M.E.M.) independently evaluated each diagnosis, without knowing the condition under which it was provided, as correct, partially correct, or incorrect (scored as 1, 0.5, or 0 points, respectively). A response was considered correct whenever it mentioned the core diagnosis, and partially correct when the core diagnosis was not cited but a constituent element of the diagnosis was mentioned. For example, in a case of gastric cancer, “Gastric malignancy” was considered correct, and “Malignancy; most likely colorectal cancer” was evaluated as partially correct. The two experts agreed in 85% of the diagnoses and solved discrepancies through discussion.

A repeated-measures ANOVA with patient socio-economic status (rich vs poor vs neutral) as within-subjects factors was performed on the mean diagnostic accuracy scores. This analysis tested the hypothesis that the description of poor patients would negatively affect diagnostic accuracy. To check whether the description of poor patients led doctors to speed up the diagnostic process, we performed a repeated-measures ANOVA with socio-economic status as a within-subjects factor on time spent to make the diagnosis. Significance levels were set at *p* < 0.05 for all comparisons. SPSS version 24.0 (SPSS Inc., Chicago, Illinois) was used for the statistical analyses.

## Results

Table 3 contains the findings from this study.

**Table 3:** Mean diagnostic accuracy scores (range 0–1; standard deviations into brackets) and mean time spent in diagnosing the written clinical cases (seconds) as a function of patients’ socio-economic status, *N* = 45

	Poor patients	Rich patients	Neutral patients
Diagnostic accuracy	0.48 (0.23)	0.52 (0.26)	0.54 (0.24)
Time spent on diagnosis	167.57 (60.01)	176.10 (65.57)	150.66 (54.08)

Participants made more mistakes when diagnosing poor patient cases relative to neutral or rich patient cases, but this effect did not reach statistical significance: *F* (2, 43) = 0.47, *p* > .05. Time spent on diagnosis did however significantly differ, *F* (2, 43) = 7.57, *p* < .01. Participants needed significantly less time to diagnose neutral patients; this effect was however entirely due to the fact that the neutral patient versions were shorter than the other cases: *F* (2, 43) = 2.27, *p* > .05.

## Discussion

Poor patients die much earlier than their more well-to-do fellow citizens [2, 3]. The question central to the present study was the extent to which doctor neglect contributes to this empirical finding. To that end, 45 residents of a Saudi medical school were presented with 12 cases in three different versions. One version described a rich patient, 1 a poor patient, and the third a patient from which no socio-economic characteristics could be deduced. Each version presented the same disease underlying the signs and symptoms presented. We failed to find any meaningful differences in diagnostic accuracy and time-to-diagnosis.

Our experiment presents a third attempt to experimentally study the influence of socio-economic status on the precision of physicians’ diagnoses and their engagement with patients of different socio-economic backgrounds, expressed as the amount of time they spent on diagnosing the cases. Two previous attempts, also failing to find differences [15, 16], had serious methodological shortcomings, and our design remediated these. The common assumption was, that doctors, perhaps due to implicit prejudices, would attach less value to the health of poorer patients and therefore would provide less appropriate care. Prejudices against poor people are broadly shared [20, 21]. Paul Gorski [22], in his “Myth of the culture of poverty” mentions a number of these preconceptions with regard to poor people: Poor people would be unmotivated and have weak work ethics; poor parents are supposed to be uninvolved in their children’s learning, largely because they do not value education; and poor people tend to abuse drugs and alcohol (all turn out to be largely false). Our participants did not seem to suffer from those prejudices: they spent equal amounts of time on each of the patients presented, and came to similar diagnostic decisions, irrespective of the socio-economic background of the patient. With some confidence, and in the light of the previous findings, we conclude that, at least within the realm of experimental approaches to the issue, doctors do not treat their patients differently based on whether they are rich or poor. We state this conclusion with some certainty because our study used a within-group design, excluding the possibility of confounding factors due to poor randomization. In addition, we have used the same methodology in similar studies, demonstrating that our procedures are sensitive to experimental manipulations of the kind attempted in this study [19, 23]. For instance, in a study on the negative influence of so called “difficult patients,” using the same methods, we were able to demonstrate rather strong effects with similarly subtle differences in the descriptions of patients [17, 18]. Finally, within-group variances are similar to those of other studies using the same methods.

A problem is the external validity of our findings. An experimental approach forces researchers to reduce complexity of the event studied, so that internal validity is assured. (Internal validity guaranties that causal conclusions drawn from the findings are accurate). However, it is possible that physicians do not so much respond to more or less “objective” indicators of poverty, such as a job that someone has, or the community where he or she comes from, but to more subtle characteristics of people in the consulting room, such as their smell, the way they talk, or the way they interact. Such characteristics cannot be studied with the approach used in this study and the ones summarized here. This implies that there is room for further study.

## Data Availability

The datasets used and/or analyzed during the current study are available from the corresponding author on reasonable request.
